# Mapping the Hidden Terrain of Hepatocellular Carcinoma: Exploring Regional Differences in Incidence and Mortality across Two Decades by Using the Largest US Datasets

**DOI:** 10.3390/jcm13175256

**Published:** 2024-09-05

**Authors:** Yazan Abboud, Vraj P. Shah, Michael Bebawy, Ahmed Al-Khazraji, Kaveh Hajifathalian, Paul J. Gaglio

**Affiliations:** 1 Department of Internal Medicine, Rutgers New Jersey Medical School, Newark, NJ 07103, USA; vs528@njms.rutgers.edu (V.P.S.); michael.bebawy@rutgers.edu (M.B.); 2 Division of Gastroenterology and Hepatology, Rutgers New Jersey Medical School, Newark, NJ 07103, USA; aa2758@njms.rutgers.edu (A.A.-K.); kh852@njms.rutgers.edu (K.H.); pjg47@njms.rutgers.edu (P.J.G.)

**Keywords:** hepatocellular carcinoma, epidemiology, geography, incidence, mortality, health disparity

## Abstract

**Background:** There is an observed variation in the burden of hepatocellular carcinoma (HCC) across different US populations. Our study aims to comprehensively assess variations in HCC incidence and mortality rates across different regions of the US. Understanding these geographical differences is crucial, given prior evidence indicating variations in the incidence of viral hepatitis and metabolic dysfunction-associated steatotic liver disease and varying access to curative HCC treatment among states. **Methods:** HCC age-adjusted incidence rates between 2001 and 2021 were obtained from the United States Cancer Statistics (USCS) database (which covers approximately 98% of the US population). HCC age-adjusted mortality rates between 2000 and 2022 were obtained from the National Center of Health Statistics (NCHS) database (covering approximately 100% of the US population). The rates were categorized by US geographical region into West, Midwest, Northeast, and South. Incidence rates were also categorized by race/ethnicity. Time trends [annual percentage change (APC) and average APC (AAPC)] were estimated by using Joinpoint Regression via the weighted Bayesian Information Criteria (*p* < 0.05). **Results:** Between 2001 and 2021, there were 491,039 patients diagnosed with HCC in the US (74.2% males). The highest incidence rate per 100,000 population was noted in the West (7.38), followed by the South (6.85). Overall incidence rates increased between 2001 and 2015 and then significantly decreased until 2021 (APC = −2.29). Most cases were in the South (38.8%), which also had the greatest increase in incidence (AAPC = 2.74). All four geographical regions exhibited an overall similar trend with an increase in incidence over the first 10–15 years followed by stable or decreasing rates. While stratification of the trends by race/ethnicity showed slight variations among the regions and groups, the findings are largely similar to all race/ethnic groups combined. Between 2000 and 2022, there were 370,450 patients whose death was attributed to HCC in the US (71.6% males). The highest mortality rate per 100,000 population was noted in the South (5.02), followed by the West (4.99). Overall mortality rates significantly increased between 2000 and 2013 (APC = 1.90), then stabilized between 2013 and 2016, and then significantly decreased till 2022 (APC = −1.59). Most deaths occurred in the South (35.8%), which also had the greatest increase in mortality (AAPC = 1.33). All four geographical regions followed an overall similar trend, with an increase in mortality over the first 10–15 years, followed by stable or decreasing rates. **Conclusions:** Our analysis, capturing about 98% of the US population, demonstrates an increase in HCC incidence and mortality rates in all geographical regions from 2000 to around 2014–2016, followed by stabilizing and decreasing incidence and mortality rates. We observed regional variations, with the highest incidence and mortality rates noted in the West and South regions and the fastest increase in both incidence and mortality noted in the South. Our findings are likely attributable to the introduction of antiviral therapy. Furthermore, demographic, socioeconomic, and comorbid variability across geographical regions in the US might also play a role in the observed trends. We provide important epidemiologic data for HCC in the US, prompting further studies to investigate the underlying factors responsible for the observed regional variations in HCC incidence and mortality.

## 1. Introduction

Primary liver cancer is the sixth most common malignancy and the third leading cause of cancer-related deaths globally, with a significant impact on global health [1]. Hepatocellular carcinoma (HCC) is the most common primary liver malignancy, accounting for 80% of all liver cancer cases [2]. HCC develops in the context of chronic liver disease and cirrhosis, conditions that result from prolonged liver injury and inflammation. The pathogenesis of HCC is complex and characterized by an interplay of genetic abnormalities, aberrant expression of non-coding RNA (ncRNA), and dysregulated epigenetic alterations, which drive malignant transformation [3]. Risk factors for HCC include chronic infections with hepatitis B virus (HBV) and hepatitis C virus (HCV), alcohol-associated liver disease (ALD), metabolic dysfunction-associated steatotic liver disease (MASLD), and exposure to dietary toxins such as aflatoxin and aristolochic acid [4,5,6]. These risk factors are largely preventable, highlighting the potential for targeted public health interventions and lifestyle modifications to reduce the incidence of HCC. Improving our understanding of the epidemiology of HCC is essential to guiding best practices to address these risk factors through vaccination programs, effective antiviral therapies, and lifestyle changes.

Globally, the incidence of liver cancer has been declining, with a significant decrease in the years 2001 to 2004; however, these global trends are largely being driven by declines in East and Southeast Asia [7]. Many countries, including the United States, continue to see steady increases in HCC. A study using data from the Surveillance, Epidemiology, and End Results (SEER) database found that both incidence and mortality rates had been increasing from 1975 to 2017 in the US [8]. Other recent studies noted that the incidence of HCC had decreased in younger populations and middle-aged populations and that overall incidence begun to plateau in 2010–2015 [9,10]. HCC incidence shows a marked male predominance, with a male-to-female ratio ranging from 2:1 to 4:1. The higher incidence may be explained by a higher prevalence of risk factors like HBV/HCV infection, alcohol use, and tobacco use in men; however a protective role of female sex hormones has also been hypothesized to contribute to those data [11].

Research looking at the geographical epidemiology in the United States is limited, with few studies being reported in the literature. One study that used the CDC WONDER database between 1999 and 2020, which is a nationally representative database of mortality rates in the US, found that HCC-related mortality was higher in urbanized areas; the study also showed that rural or less densely populated areas experienced an increase in age-adjusted mortality [12]. Another study evaluating patients admitted with a primary or secondary diagnosis of HCC showed that the most common geographical location was in the South region, accounting for 36% of HCC-related US hospitalizations [13].

While chronic HBV or HCV infection remains the most common etiology for HCC worldwide, the implementation of vaccine programs and advancements in antiviral agents have contributed to an epidemiologic shift with decreases in cases secondary to HBV and HCV and increased cases due to ALD and MASLD [2,7,14]. However, further research is needed to evaluate the geographical variations in the prevalence and rates of HCC in the US and thus guide future population-specific interventions and resource allocation to effectively address the burden of HCC. Our study aims to comprehensively assess how HCC incidence and mortality rates vary across regions of the US. Understanding these geographical differences is crucial, given prior evidence indicating variations in the incidence of HBV, HCV, ALD, and MASLD when comparing US regions. By analyzing regional trends, we hope to identify areas with the highest burden of HCC and tailor public health interventions to reduce the impact of this devastating disease.

## 2. Methods

The incidence rates of HCC between 2001 and 2021 were obtained from the United States Cancer Statistics (USCS) database. The USCS is a nationally representative database that covers approximately 98% of the US population, and it is the official source of cancer statistics in the US [15]. The USCS database integrates data from two national databases: the National Program of Cancer Registries (NPCR) by the CDC and the SEER database by the NCI. These programs collectively provide comprehensive coverage of all 50 US states, the District of Columbia, and Puerto Rico. Post-collection, rigorous processes ensure that the data adhere to the high standards set by the North American Association of Central Cancer Registries’ Data Standards, maintaining consistency and quality [16].

The mortality rates of HCC between 2000 and 2022 were obtained from the CDC’s National Center for Health Statistics (NCHS) database. The NCHS database is the most comprehensive source of cancer mortality statistics in the US and covers nearly 100% of the US population [17]. Mortality data in the NCHS are sourced from the National Vital Statistics System, which gathers information on births and deaths from vital registries across the United States. Data collection is performed via an electronic death registration system that automates data capture and documentation. Causes of death are classified according to death certificates and the International Classification of Diseases (ICD), adhering to World Health Organization guidelines. Continuous monitoring ensures data quality is upheld across all stages of data collection and processing.

HCC incidence and mortality rates per 100,000 population were age-adjusted to the standard 2000 US population by using SEER*Stat software (v.8.4.3; National Cancer Institute (“NCI”), Bethesda, Maryland, USA). The analysis included tumors with malignant behavior only. The rates were categorized by US geographical region into West, Midwest, Northeast, and South. Incidence rates were also categorized by race/ethnicity into several groups as reported in the database: Non-Hispanic White (NHW), Non-Hispanic Back (NHB), Hispanic (H), Non-Hispanic Asian/Pacific Islander (API), and Non-Hispanic American Indian/Alaskan Native (AIAN). Time trends were reported as annual percentage change (APC), which reflects the change in rates between two subsequent years, and average APC (AAPC), which reflects the average change between the rates over the entire study period. The trends were generated by using Joinpoint Regression Software (v.5.2.0.0; NCI) via the weighted Bayesian Information Criteria “BIC” methodology, which is preferred given its flexibility and superior performance across different analytical scenarios [18,19,20]. In our regression model, the age-adjusted rates were the dependent variable, while the year of diagnosis was chosen as the independent variable. The trends were evaluated by using parametric estimations utilizing a two-sided *t*-test and a *p*-value cutoff of 0.05.

## 3. Results

### 3.1. Incidence Rates and Trends

Between 2001 and 2021, there were 491,039 patients diagnosed with HCC in the US with an overall age-adjusted incidence rate of 6.58 per 100,000 population. Male patients represented 74.2% of the cases with an overall incidence rate of 10.42 per 100,000 population, compared with female patients, with an incidence of 3.19 per 100,000 population. Most cases were diagnosed in the South (190,600 patients; 38.8% of all cases) followed by the West (121,509 patients; 24.6%), the Northeast (90,668 patients; 18.5%), and lastly, the Midwest (88,262 patients; 18.0%). The highest overall incidence rate per 100,000 population over the study period was noted in the West (7.38), followed by the South (6.85), the Northeast (6.38), and the Midwest (5.39).

**Figure 1:**
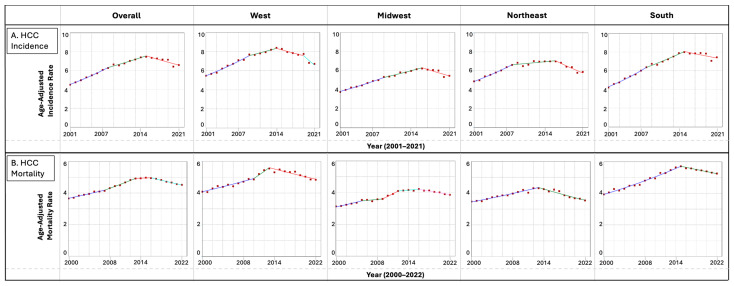
(**A**) Time trends and age-adjusted incidence rates per 100,000 population for hepatocellular carcinoma (HCC) categorized by US geographical region between 2001 and 2021. (**B**) Time trends and age-adjusted mortality rates per 100,000 population for hepatocellular carcinoma (HCC) categorized by US geographical region between 2000 and 2022.

Overall incidence rates per 100,000 population significantly increased from 4.50 in 2001 to 7.46 in 2015 and then significantly decreased to 6.59 in 2021 (APC = −2.29) (Table 1). In the West, incidence rates per 100,000 population mirrored the overall population and were observed to increase significantly from 5.47 in 2001 to 8.39 in 2014, followed by a stable trend between 2014 and 2019 and a significantly decrease in incidence to 6.71 in 2021 (APC = −6.70). In the Midwest, incidence rates per 100,000 population increased from 3.76 in 2001 to 5.33 in 2009 (APC = 4.12), then stabilized between 2009 and 2016, and then significantly decreased from 6.22 in 2016 to 5.46 in 2021 (APC = −2.89). In the Northeast, incidence rates per 100,000 population mirrored the overall population and increased from 4.88 in 2001 to 6.99 in 2016 and then significantly decreased to 5.88 in 2021 (APC = −3.96). Lastly, in the South, the trends also mirrored the overall population, with the greatest significant increase in incidence rates per 100,000 population from 5.47 in 2001 to 8.29 in 2015 followed by a significant decrease to 6.71 in 2021 (APC = −1.49) (Figure 1A).

When evaluating race/ethnic-specific rates and trends of HCC, there were variations among US geographical regions. In the West, NHW, NHB, H, and AIAN individuals followed a similar trend with an initial increase (APCs *p* < 0.05) followed by a significant decrease or stable trend in recent years (Table 2). API individuals had a stable trend followed by a significant decrease starting in 2007. For the Midwest, similar findings to the West region were seen in NHW, NHB, and API individuals, with a decrease in HCC incidence in recent years (APCs *p* < 0.05). However, for Hispanics (5074 patients), HCC incidence steadily increased between 2001 and 2021 (APC = 1.07; *p* = 0.01). For the Northeast, the rates initially increased then stabilized or decreased in recent years among NHW, NHB, and H individuals. For API individuals, HCC rates steadily decreased between 2001 and 2021 (AAPC = −2.92; *p*< 0.001). Lastly, in the South, similar findings were seen with an initial increase followed by a decline or stabilizing of rates among NHW, NHB, H, and AIAN individuals. For API individuals, HCC rates were stable until 2019 and then decreased until 2021 (APC = −10.59; *p*< 0.001).

### 3.2. Mortality Rates and Trends

Between 2000 and 2022, there were 370,450 patients in the US whose death was attributed to HCC, with an overall age-adjusted mortality rate of 4.54 per 100,000 population. Male patients represented 71.6% of the deaths with an overall mortality rate of 7.17 per 100,000 population, compared with female patients, who had a mortality rate of 2.36 per 100,000 population. Most deaths occurred in the South (132,484 deaths; 35.8% of all deaths) followed by the West (83,667 deaths; 22.6%), the Midwest (65,606 deaths; 17.7%), and lastly, the Northeast (58,494 deaths; 15.8%). The highest overall mortality rate per 100,000 population over the study period was noted in the South (5.02), followed by the West (4.99), the Northeast (3.93), and the Midwest (3.84).

Overall mortality rates per 100,000 population were noted to have significantly increase from 3.66 in 2000 to 4.92 in 2013 (APC = 1.90); the rates then stabilized between 2013 and 2016 and then decreased from 4.95 in 2016 to 4.52 in 2022 (APC = −1.59) (Table 1). In the West, mortality rates were stable between 2000 and 2013 and then significantly decreased from 5.54 in 2013 to 4.84 in 2022 (APC = −1.53). In the Midwest, there were variations in the trends, with an overall increasing or stable trend between 2000 and 2016, followed by a significantly decrease in mortality from 4.22 in 2016 to 3.86 in 2022 (APC = −1.35). In the Northeast, mortality rates per 100,000 population significantly increased from 3.48 in 2000 to 4.31 in 2013 (APC = 1.82), then significantly decreased to 3.54 in 2022 (APC = −2.23). In the South, mortality rates per 100,000 population experienced the greatest increase from 3.93 in 2000 to 5.71 in 2015 (APC = 2.53) and then significantly decreased to 5.26 in 2022 (APC = −1.21) (Figure 1B).

## 4. Discussion

Our nationwide analysis of two databases, which accounted for nearly all patients with HCC in the US, demonstrated an increase in both HCC age-adjusted incidence and mortality for approximately 10–15 years from the year 2000, followed by stabilization or decreasing trends until 2021–2022. We appreciated overall similar trends across all geographical regions in the US, encompassing the South, Northeast, West, and Midwest regions. However, we observed notable inter-regional differences. While the highest incidence and mortality rates were noted in the South and West, patients with HCC in the South experienced the greatest increase in incidence and mortality across all geographical regions.

These findings are consistent with previously published epidemiological trends for the incidence of HCC in the US, which have demonstrated increasing incidence up until 2014, followed by a stable or declining trend [10,21]. Our study expands on previously published studies by extending our analysis until 2021, whereas the existing literature only includes incidence data until around 2017–2018 [22,23]. Furthermore, our data enhance previously published literature by stratifying the HCC burden by geography and showing the variations in the trends across different regions in the US [24,25]. The shift in HCC incidence which began around 2014–2016 is likely attributed to a variety of factors. The observed rates of HCC coincide with the development and increased utilization of direct-acting antiviral (DAA) therapy for chronic HCV [25]. The first DAA therapy was approved for use in the US in 2011, with a significant acceleration in the incorporation of DAA therapy around 2013–2014 with cure rates of chronic HCV reaching 100% [26,27]. Aside from the introduction of DAA therapy at that time, the observed decrease in incidence could also be attributed to ongoing HBV vaccinations and other more effective antiviral therapy to control HBV replication [28]. Another potential explanation regarding the recently observed changes in HCC incidence is the potential impact of the COVID-19 pandemic during 2020 and 2021 leading to missed clinic appointments and diminished surveillance rates, resulting in underdiagnosing HCC.

Across the various regions within the US, our study demonstrated that the highest incidence of HCC was in the West region, closely followed by the South region. Although the nature of the databases used for our analysis limits further investigation into the underlying cause of these differences, a variety of factors are likely contributing to the observed regional variations. Specifically, the West and South regions have larger Hispanic populations, which have previously been demonstrated to have a higher incidence of HCC when compared with other racial/ethnic groups [29,30]. Furthermore, the West region has a high Asian American population, another racial/ethnic subgroup that has also been previously shown to have higher incidences of HCC [31,32]. Of note, the high incidence of HCC in the South region can also further be attributed to the fact that this region has disproportionately high rates of obesity and metabolic syndrome [33].

When evaluating risk factors for liver disease and HCC, alcohol intake has been shown to be independently associated with cirrhosis development and HCC, especially in patients with chronic HCV infection, and it is also associated with increased mortality in those with HCV or HBV infections [34]. When evaluating alcohol use, according to 2022 data from the Centers for Disease Control and Prevention (CDC), the Midwest and the South have higher rates of alcohol binge drinking, with the Northeast having the lowest rates [35]. Furthermore, individuals in the South and the West were found to be at higher odds of having alcohol-related deaths [36]. Additionally, there has been a steady increase in ethanol use across the US from 2000 to 2021. This increase in alcohol intake with the variations in burden among US regions could result in an increase in incidence and mortality of HCC and likely contribute to the geographical variations seen in our study [37].

Additionally, multiple recent studies have investigated the impact of urban versus rural settings for the incidence of HCC. A majority of studies have found that although urban areas have a higher incidence rate of HCC, there has been a recent shift, with rural communities now having a greater increasing incidence [38,39,40]. Given that the various regions of the US have varying distributions of urban and rural areas, it is likely that such differences are also contributing to the regional variation observed in HCC incidence. As HCC has a multitude of risk factors, further studies are required to better elucidate the geographical variation observed in our study.

We also observed that the decline in HCC incidence after 2014–2016 was most prominent in the West and the Northeast, whereas the South had the least prominent decline. It is possible that the rapid decline seen in the West and the Northeast is due to a combination of a high proportion of insured patients and increased access to physicians per capita [41]. It is possible that patients residing in those regions were more likely to receive DAA therapy soon after its introduction when compared with those living in the South and the Midwest. Furthermore, the South region has the highest rates of uninsured individuals, potentially leading to patients being less likely to receive DAA therapy, thus resulting in a more gradual decline in HCC incidence [41]. Furthermore, the lower socioeconomic status of the population in the South, coupled with limited access to healthcare, often results in high rates of loss of follow-up among patients with chronic liver disease. This lack of continuity in care can contribute to the progression of the disease to HCC. Our data also provide further information on the regional change of HCC incidence among different race/ethnic groups in the US.

Recent epidemiological changes in chronic liver disease and cirrhosis might also contribute to the geographical and temporal variation observed in our study. Specifically, MASLD now contributes to a large proportion of HCC cases in the US and is the most rapidly growing contributor to liver morbidity and mortality [42]. Of note, Hispanic patients, women, patients above the age of 50, and those experiencing food insecurity have had the largest increase in MASLD and fibrosis incidence in the US in the past few years [43]. As such, this would also contribute to the South region having a disproportionately higher incidence of HCC. Furthermore, prior nationwide data showed disparities in the mortality rates and trends of chronic liver disease and cirrhosis in the US, with variation between US states and patient populations [44]. The study showed an increase in mortality rates from chronic liver disease and cirrhosis in older and younger men and women, more pronounced in younger women when compared with counterpart men. These findings suggest an ongoing essential need to further investigate the contributions leading to this increase in mortality given its likely association with HCC outcomes.

Similar to our findings on HCC incidence, we observed that age-adjusted mortality for HCC in the US increased from 2000 to around 2013–2015 and then began to stabilize and eventually decrease. The same general trend was also appreciated in all four regions of the US. Overall, the recent decrease in mortality is likely multifactorial [45,46]. As an example, increased screening for HCC in high-risk patients has resulted in greater early diagnosis for patients, which then leads to overall prolonged survival and decreased mortality rates [47,48,49]. In addition to early diagnosis, there continue to be advances in therapies with also increased access to care. Specifically, there have been increased numbers of liver transplantations and hepatic resections and increased utilization of loco-regional therapy, such as radiofrequency ablation and transarterial chemoembolization, as well as introduction of chemotherapeutic and immunologic agents for the treatment of HCC [50,51,52,53,54].

Upon regional analysis, the South and West regions had the highest mortality rates. Similar to the factors contributing to the increased incidence in these regions, it is likely that racial/ethnic differences, poor access to care, socioeconomic variability, and underlying disease contributed to these observed differences [55]. Specifically, patients in the South might have higher rates of mortality due to increased rates of obesity, metabolic disorder, alcohol use, hepatitis C prevalence, and poor access to care [56]. The greatest decline in mortality was observed in the Northeast region, which could be due to increased access to care [41].

It is estimated that around 25% of the United States population have MASLD [57]. Given that a significant percentage of patients without known liver disease actually have fibrotic and/or cirrhotic changes, there has also been a recent increase in screening and surveillance for liver disease [58]. As such, patients of higher socioeconomic status are more likely to be insured and undergo such screenings and surveillance. This early detection could also possibly contribute to decreased mortality in higher socioeconomic patients. Recent innovations have also likely contributed to decreased mortality, with new approaches such as percutaneous treatment and trans-arterial techniques [58].

Of note, there continues to be an international and national push towards better surveillance for HCC and liver disease; current surveillance rates for both primary care physicians and gastroenterologists are suboptimal [59,60]. Further investigation is required to elucidate the impact of this increased emphasis on screening and surveillance. Although HCC surveillance used to be primarily ultrasound-based, several imaging modalities are now utilized, especially multi-phase computed tomography (CT) and contrast-enhanced magnetic resonance imaging in patients with positive surveillance tests [61]. However, regional differences exist in the access to and utilization of imaging machines. Specifically, patients in rural settings are less likely to receive appropriate imaging than patients in urban settings [62]. This would correlate with patients in the Midwest and the South, who are more likely to reside in rural regions. Although there is a paucity of information on MRI machine distribution by region in the US, current data demonstrate that the South and the West have the lowest number of MRI technologists per capita [63]. These data can be used as a proxy to reflect patient access to MRI machines, and as such, patients in these regions would be less likely to receive appropriate imaging, which could delay treatment and result in increased mortality.

Socioeconomic status might also contribute to the regional differences appreciated in our study, as these patients are more likely to be able to afford treatments such as immune checkpoint inhibitors. This might be one of the contributions leading to higher mortality in the South given the historically lower socioeconomic status of patients in that region. Additionally, patients with poorer access to healthcare are less likely to receive such screenings and thus more likely to have advanced disease and increased mortality. This is consistent with the fact that individuals in the South have poor access to care and have higher mortality, whereas individuals in the Northeast have increased access to care and decreased mortality. However, the further monitoring of these trends is required, given recent therapies such as metronomic capecitabine, which is both efficacious and relatively affordable [64,65].

Our findings are relatively consistent with previously published studies in other Western countries, including Italy [66]. That study grouped patients by year of HCC diagnosis into 2000–2004, 2005–2009, and 2010–2014. Similar to our study, the authors demonstrated an increase in incidence from 2000 to 2014. This study also investigated trends in the etiology of HCC and found an increase in the incidence of non-viral, MASLD, and cryptogenic causes of HCC. They also reported an increase in surveillance for HCC over time, which might contribute to the increase in incidence that they found. Increased surveillance also leads to the detection of early disease and overall decreasing mortality. While the study in Italy found a decrease in mortality between 2000 and 2014, we reported an increase in mortality from 2000 to around 2014 and then a decrease in mortality afterward. It is possible that differences in screening, HCC etiology, and healthcare policy contributed to these observed differences.

Although HCC continues to pose a significant burden on patients in the US, there is currently a paucity of research investigating geographical differences and trends for both HCC incidence and mortality rates. Our study contributes to the existing literature, by providing a robust analysis of regional trends within the US across a 20-year time period. We also provide data on the regional changes in HCC incidence per race/ethnic group. Furthermore, we utilized the USCS and NCHS databases to provide a nearly complete representation of all cases within the US during the time period that was analyzed. However, as with any study utilizing national databases, our study is limited by the nature of these databases. Specifically, our analysis is limited by the lack of clinical variables associated with HCC diagnosis and progression. As a result, we were unable to parse out the underlying factors responsible for the novel findings from our study. Stratifying the etiology of cirrhosis and subsequent HCC would provide valuable insights into the revealed regional variations in HCC incidence and mortality and warrants investigation in future studies. In addition, stratifying HCC based on the status of prior cirrhosis would also help to better understand the revealed variations and needs future investigations. As with any database, there is also the possibility of inaccurate coding or loss of records which could alter our findings. A prior study by Park et al. has discussed several limitations of the SEER database [67]. We utilized the USCS database, which is composed of the NPCR and SEER databases; thus, the limitations seen in SEER database can also be seen in the USCS database. These include the possibility of miscoding, which has been noted in several cancers and populations. Furthermore, other limitations include the possibility of over- or under-reporting certain cancers due to the migration of patients between different states that report data to either the SEER program or NPCR. In order to minimize such errors, each of the three databases utilized in our study has a robust process to ensure data validity. There are several quality checks and verification steps that occur prior to the publication of cancer data to maintain high standards. We opted to utilize national databases for this study, as they are the most effective and accurate in characterizing national trends over time and across geographical regions. We specifically used Joinpoint Regression for time trend analysis via the modified Bayesian Information Criteria methods, as this statical method is recommended to detect trend changes, especially when using nationwide databases. Future studies should evaluate the future forecasting of the incidence and mortality rates and trends of HCC in different regions of the US, which can be informative for public health.

Ultimately, our nationwide analysis, which captures about 98% of the US population, demonstrated an increase in HCC incidence and mortality rates in all geographical regions from 2000 to around 2014–2016, followed by stabilizing and decreasing incidence and mortality rates. Moreover, we observed regional variations, with the highest incidence and mortality rates in the West and South regions. The South experienced the fastest increase in both incidence and mortality related to HCC over the first 15 years of the study and the slowest decrease afterward. These findings are likely explained by the introduction of DAA therapy, improved HBV therapies, and the demographic, socioeconomic, and comorbid variability across geographical regions in the US. Overall, our study provides crucial epidemiologic data for HCC in the US, prompting further studies to investigate the underlying factors responsible for the observed regional variation in HCC incidence and mortality.

## Figures and Tables

**Table 1:** Time trends of hepatocellular carcinoma (HCC) incidence rates between 2001 and 2021 and mortality rates between 2000 and 2022 in different geographical regions in the US.

Geographical Location	Sample Size	Trends ^c^				
		Time Period	APC (95% CI)	*p*-Value	AAPC (95% CI)	*p*-Value
Incidence Data between 2001 and 2021 ^a^						
**All United States**	491,039 (100%)	2001–2008	4.88 * (4.11 to 7.44)	<0.001	1.88 * (1.66 to 2.21)	<0.001
		2008–2015	2.58 * (0.91 to 3.38)	0.02		
		2015–2021	−2.29 * (−3.40 to −1.47)	0.005		
**West**	121,509 (24.7%)	2001–2009	4.19 * (2.87 to 7.09)	0.005	0.95 * (0.71 to 1.27)	<0.001
		2009–2014	1.94 * (0.53 to 5.23)	0.01		
		2014–2019	−1.91 (−2.66 to 1.36)	0.08		
		2019–2021	−6.70 * (−9.24 to −3.56)	<0.001		
**Midwest**	88,262 (18.0%)	2001–2009	4.12 * (3.45 to 7.30)	<0.001	1.79 * (1.54 to 2.12)	<0.001
		2009–2016	2.58 (−1.12 to 3.25)	0.07		
		2016–2021	−2.89 * (−4.66 to −1.71)	0.01		
**Northeast**	90,668 (18.5%)	2001–2008	4.52 * (3.70 to 5.83)	<0.001	0.85 * (0.60 to 1.14)	<0.001
		2008–2016	0.78 * (0.03 to 1.53)	0.04		
		2016–2021	−3.96 * (−5.57 to −2.93)	<0.001		
**South**	190,600 (38.8%)	2001–2008	5.79 * (4.71 to 10.48)	<0.001	2.74 * (2.45 to 3.20)	<0.001
		2008–2015	3.44 * (0.16 to 4.31)	0.04		
		2015–2021	−1.49 * (−2.95 to −0.47)	0.02		
Mortality Data Between 2000 and 2022 ^b^						
**All United States**	370,450 (100%)	2000–2007	1.90 * (1.17 to 2.17)	<0.001	0.95 * (0.88 to 1.01)	<0.001
		2007–2013	2.77 * (2.49 to 3.45)	<0.001		
		2013–2016	0.25 (−0.68 to 1.44)	0.40		
		2016–2022	−1.59 * (−1.91 to −1.39)	<0.001		
**West**	83,667 (22.6%)	2000–2010	1.88 (−0.12 to 2.44)	0.05	0.81 * (0.63 to 1.00)	<0.001
		2010–2013	4.45 (−2.15 to 5.52)	0.09		
		2013–2022	−1.53 * (−2.10 to −0.74)	0.02		
**Midwest**	65,606 (17.7%)	2000–2005	2.34 * (1.82 to 3.72)	<0.001	1.02 * (0.93 to 1.14)	<0.001
		2005–2009	0.88 (−0.12 to 1.57)	0.08		
		2009–2012	4.41 * (3.02 to 5.16)	0.001		
		2012–2016	0.62 (−0.52 to 1.37)	0.15		
		2016–2022	−1.35 * (−1.88 to −0.98)	0.004		
**Northeast**	58,494 (15.8%)	2000–2013	1.82 * (1.51 to 2.17)	<0.001	0.14 (−0.01 to 0.31)	0.06
		2013–2022	−2.23 * (−2.77 to −1.74)	<0.001		
**South**	132,484 (35.8%)	2000–2015	2.53 * (2.37 to 2.73)	<0.001	1.33 * (1.23 to 1.44)	<0.001
		2015–2022	−1.21 * (−1.65 to −0.76)	<0.001		

^a^ Data are presented as the number of HCC cases followed by percentages of the number of HCC patients from the total cases of HCC in the database. ^b^ Data are presented as the number of deaths followed by percentages of the number of deaths from the total deaths of HCC in the database. ^c^ Time trends were computed by using Joinpoint Regression Program (v5.2.0.0; NCI, Bethesda, Maryland, USA) with 3 maximum joinpoints allowed (4-line segments). * Implies statistical significance.

**Table 2:** Time trends of hepatocellular carcinoma (HCC) incidence rates between 2001 and 2021 in different geographical regions in the US categorized by race/ethnicity.

Geographical Location	Sample Size ^a^	Trends ^b^				
		Time Period	APC (95% CI)	*p*-Value	AAPC (95% CI)	*p*-Value
**West**						
**NHW**	59,743 (12.2%)	2001–2009	5.04 * (4.61 to 5.83)	<0.001	1.52 * (1.36 to 1.71)	<0.001
		2009–2014	2.61 * (1.53 to 3.59)	<0.001		
		2014–2019	−1.77 * (−2.37 to −0.79)	<0.001		
		2019–2021	−6.33 * (−7.96 to −4.23)	<0.001		
**NHB**	7202 (1.5%)	2001–2013	3.30 * (1.54 to 6.15)	0.02	−0.47 (−0.96 to 0.16)	0.11
		2013–2018	−3.69 (−5.31 to 4.84)	0.24		
		2018–2021	−9.40 * (−14.92 to −5.49)	<0.001		
**H**	30,650 (6.24%)	2001–2014	3.04 * (2.32 to 4.10)	<0.001	0.93 * (0.49 to 1.44)	0.001
		2014–2021	−2.87 * (−4.78 to −1.50)	<0.001		
**API**	20,720 (4.2%)	2001–2007	0.07 (−1.42 to 5.35)	0.81	−2.62 * (−3.03 to −2.16)	<0.001
		2007–2015	−2.18 * (−7.70 to −1.14)	0.01		
		2015–2021	−5.81 * (−0.10 to −3.02)	0.006		
**AIAN**	2821 (0.6%)	2001–2015	5.34 * (4.02 to 7.80)	<0.001	2.37 * (1.30 to 3.61)	<0.001
		2015–2021	−4.23 * (−10.37 to −0.90)	0.01		
**Midwest**						
**NHW**	64,592 (13.2%)	2000–2016	3.34 * (3.01 to 3.72)	<0.001	1.72 * (1.44 to 1.98)	<0.001
		2016–2021	−2.98 * (−4.92 to −1.62)	<0.001		
**NHB**	13,863 (2.8%)	2000–2008	5.01 * (3.65 to 11.21)	<0.001	0.74 * (0.32 to 1.27)	0.003
		2008–2015	2.26 (−4.14 to 3.33)	0.14		
		2015–2021	−5.69 * (−7.83 to −4.19)	0.002		
**H**	5074 (1.0%)	2001–2021	1.07 * (0.21 to 2.17)	0.01	1.07 * (0.21 to 2.17)	0.01
**API**	3065 (0.6%)	2000–2016	−0.33 (−1.29 to 16.17)	0.92	−1.57 (−2.79 to 0.60)	0.06
		2016–2022	−5.23 * (−16.99 to −1.40)	0.006		
**AIAN**	905 (0.2%)	**				
**Northeast**						
**NHW**	57,641 (11.7%)	2000–2008	4.13 * (3.27 to 6.12)	<0.001	1.10 * (0.82 to 1.42)	<0.001
		2008–2016	1.11 * (0.12 to 1.98)	0.03		
		2016–2021	−3.00 * (−5.10 to −1.85)	<0.001		
**NHB**	13,066 (2.7%)	2000–2009	5.56 * (4.43 to 7.37)	<0.001	−0.14 (−0.56 to 0.32)	0.57
		2009–2016	−0.23 (−1.75 to 1.18)	0.72		
		2016–2021	−8.49 * (−11.05 to −6.77)	<0.001		
**H**	12,009 (2.5%)	2001–2006	5.95 * (1.16 to 18.08)	0.03	0.07 (−0.60 to 1.10)	0.77
		2006–2014	−0.14 (−4.94 to 5.62)	0.88		
		2014–2021	−3.70 * (−9.70 to −0.76)	0.03		
**API**	7232 (1.5%)	2000–2016	−0.98 * (−1.60 to −0.11)	0.02	−2.92 * (−3.53 to 2.37)	<0.001
		2016–2022	−8.52 * (−12.78 to −6.02)	<0.001		
**AIAN**	216 (0.04%)	**				
**South**						
**NHW**	115,927 (23.6%)	2000–2014	4.85 * (4.33 to 5.51)	<0.001	2.86 * (2.56 to 3.19)	<0.001
		2014–2021	−0.74 (−1.92 to 0.25)	0.13		
**NHB**	36,188 (7.4%)	2000–2008	7.16 * (5.63 to 12.32)	<0.001	2.14 * (1.73 to 2.76)	<0.001
		2008–2015	2.94 * (0.39 to 4.39)	0.03		
		2015–2021	−4.28 * (−6.23 to −2.96)	0.002		
**H**	29,532 (6.0%)	2001–2016	2.19 * (1.71 to 3.07)	<0.001	1.12 * (0.67 to 1.62)	<0.001
		2016–2021	−2.00 * (−5.39 to −0.19)	0.03		
**API**	6572 (1.3%)	2000–2014	−0.93 (−3.87 to 6.15)	0.26	−2.71 * (−3.12 to −1.91)	<0.001
		2014–2019	−3.99 (−5.36 to 1.30)	0.13		
		2019–2021	−10.59 * (−14.98 to −5.22)	<0.001		
**AIAN**	1591 (0.3%)	2001–2004	22.64 * (3.72 to 72.59)	0.009	5.10 * (3.25 to 8.18)	<0.001
		2004–2018	3.87 * (1.17 to 10.20)	0.03		
		2018–2021	−4.80 (−17.71 to 2.73)	0.19		

^a^ Data are presented as the number of HCC cases followed by percentages of the number of HCC patients from the total cases of HCC in the database. ^b^ Time trends were computed by using Joinpoint Regression Program (v5.2.0.0; NCI) with 3 maximum joinpoints allowed (4-line segments). * Implies statistical significance. ** There were too few cases in at least one calendar year to estimate a trend.

## Data Availability

The findings of this study were accepted in part at the American College of Gastroenterology Annual Scientific Meeting & Postgraduate Course.
